# Editorial: Sports nutrition: muscle adaptation via supplementation and other nutritional strategies

**DOI:** 10.3389/fnut.2024.1539316

**Published:** 2024-12-19

**Authors:** Dillon R. Harris, Thomas D. Cardaci, Ahmed Ismaeel, David D. Church, Jeffery L. Heileson, Steven B. Machek

**Affiliations:** ^1^Department of Kinesiology and Sport Management, Texas A and M University, College Station, TX, United States; ^2^Department of Pathology, Microbiology, and Immunology, University of South Carolina School of Medicine, Columbia, SC, United States; ^3^Department of Physiology, College of Medicine, University of Kentucky, Lexington, KY, United States; ^4^University of Arkansas for Medical Sciences, Little Rock, AR, United States; ^5^Walter Reed National Military Medical Center, Bethesda, MD, United States; ^6^California State University, Monterey Bay, Seaside, CA, United States

**Keywords:** skeletal muscle, ergogenic aids, nutraceuticals, dietary strategies, supplementation

Skeletal muscle is essential for voluntary movement and metabolic health, especially considering age-associated muscle mass and accompanying functional deficits that may significantly impact our daily independence and overall quality of life (1). Beyond its fundamental role in facilitating locomotion, skeletal muscle's plasticity in response to various stimuli is well-documented (2–4). Alterations occur from the molecular to the whole-muscle level potentially improving function and enhancing performance (5). While mechanical tension is a key stimulus for skeletal muscle adaptation, nutritional strategies can often bolster these adaptations by more effectively improving muscle growth and subsequent function (6, 7). Further, there are many novel ergogenic aids (substance used for the purpose of enhancing performance) and other nutritional strategies that purportedly augment performance metrics spanning muscular strength, aerobic capacity, and even clinical improvements in function capacity (3, 8, 9). By exploring various nutritional factors this Research Topic explores how emerging compounds and dietary modifications may influence skeletal muscle's adaptation to exercise and performance enhancement.

Whole plants and their extracts have been widely acknowledged for their ability to potentiate several essential biological properties, including anti-inflammatory, anabolic/catabolic modulation, cardioprotective effects, and adaptogenic benefits (9). Lee et al. explored the effects of *Astragalus membranaceus* and *Paeonia japonica* (APX) in a weighted-treadmill rodent exercise model. Animals supplemented with APX exhibited enhanced muscle growth without altering body weight or fat mass when compared to exercise alone. Mechanistic investigations by Lee et al. in APX-treated C2C12 myoblasts and in tissues of exercised-mice suggest that APX may promote hypertrophy by regulating myogenic transcription factors (e.g., MyoD and myogenin). Building upon that premise that mechanical tension is a key driver of hypertrophy, Salter et al. assessed how daily supplementation with *Sphaeranthus indicus* and *Mangifera indica* extracts (SMI) might enhance the strengthening effects of resistance exercise training (RET). To investigate the former, the authors recruited 120 apparently healthy men to undergo a randomized, double-blind, placebo-controlled study. After 8 weeks, RET and SMI supplementation significantly improved bench press and leg extension strength, as well as repetitions to failure (RTF) when compared to RET alone. The increased testosterone and lowered cortisol concentrations were observed specifically in the groups receiving SMI supplementation, suggesting its role in optimizing anabolic conditions for muscle growth. Together, these studies highlight the potential of whole plant extracts to enhance muscle adaptations to exercise and promote overall muscle health.

Sarcopenia is a complex syndrome commonly characterized by age-related reductions in muscle mass, deteriorated muscular strength, and diminished functional capacity (10). As the population ages, understanding the biomarkers linked to these declines in skeletal muscle health has become a significant area of interest and importance. Xie et al. highlighted that serum klotho levels, a novel beta-glucuronidase biomarker, can be a potential marker in addressing age-related declines in skeletal muscle mass. Specifically, their cross-sectional investigation found that higher serum klotho levels are inversely associated with the risk of low muscle mass in middle-aged adults, wherein this relationship being particularly evident in women. Alongside identifying biomarkers, nutritional interventions focusing on essential amino acids (EAAs) have gained attention for their role in combating this age-related loss of muscle mass. Although the lower limit of effective EAA dosing has yet to be elucidated, Church et al. demonstrated that 3.6 g of a high-leucine composition plus arginine significantly improves muscle protein fractional synthesis rate (FSR) in older adults. Specifically, muscle protein FSR increased by 0.058%/hour over a 3-h period following consumption and when accounting for total muscle mass this increase in FSR represented approximately 80% of the ingested EAAs. These findings by Xie et al. and Church et al. highlight the potential of both biomarkers and targeted nutritional interventions (e.g., low doses of EAAs) to support muscle health especially in the context of sarcopenia.

Often overshadowed by their macronutrient counterparts, micronutrients are crucial in skeletal muscle adaptation by supporting cellular processes that are essential for optimizing muscle growth, recovery, and overall function (11). Without key micronutrients, such as iron and vitamin B6, impairments to athletic performance can occur due to disruptions in energy metabolism, oxygen transport, and reduced exercise capacity. However, the underlying mechanisms responsible for these effects are not fully understood. Consequently, the investigation by Zhou et al. reported that long-term (e.g., 30 days) iron supplementation combined with vitamin B6 enhances mitochondrial function rescues mitochondrial activity under adverse conditions. More specifically, the authors suggest that through its effects on Complex I- and Complex II-driven ATP production, iron and vitamin B6 can increase VO_2_ max by positively influenced mitochondrial biogenesis as well as metabolism in skeletal muscle. Zhou et al.'s findings highlight micronutrient roles toward supporting muscular bioenergetics and the associated metabolic pathways that are essential for exercise-mediated skeletal muscle adaptation.

The studies discussed in this Research Topic cumulatively provide critical insights into nutritional strategies - such as EAAs and plant-derived compounds like *Astragalus membranaceus*- toward synergistically promoting skeletal muscle adaptations concurrent with exercise. Furthermore, novel biomarkers like serum klotho are promising prospective biomarkers to monitor and address age-related declines in muscle health. While the current literature is rich with data regarding strategies to improve broad-ranging skeletal muscle-associated characteristics (12, 13), the investigations highlighted within this topic illustrate that there is a wealth of nutritionally-focused strategies we have yet to employ that may benefit both clinical and athletic populations.
